# Education Research: A Behavioral Intervention to Improve Group-Based Diagnostic Quality and Educational Experience Among Neurology Trainees: A Feasibility Study

**DOI:** 10.1212/NE9.0000000000200216

**Published:** 2025-04-09

**Authors:** Jeremy Ader, Isaac Raymundo, Adam D. Galinsky, Modupe Akinola, Michelle Bell

**Affiliations:** 1 Department of Neurology, NYU Grossman School of Medicine, New York, NY;; 2 Management Division, Columbia Business School, New York, NY; and; 3 Department of Neurology, Columbia University Irving Medical Center, New York, NY.

## Abstract

**Background and Objectives:**

“Brain-writing” is a technique in which group members write down ideas individually, before a group discussion, to improve idea generation and individual engagement in group discussions. We assessed the feasibility of studying the impact of brain-writing on diagnostic quality and educational experience among neurology residents in a small case-based learning environment.

**Methods:**

We conducted a repeated-measures study, conducted over 6 sessions consisting of groups of 3 to 5 neurology residents from different years of training. During each session, 3 cases were treated as control, “brainstorming,” cases, and 3 were intervention, “brain-writing,” cases, in which the group wrote down possible diagnoses and tests before engaging in a group discussion. Tests and diagnoses from the brain-writing exercise and group discussion as well as a post case survey on participant experience were recorded through a Qualtrics survey, and video recordings were reviewed to determine speaking order and number of tests and diagnoses verbalized by each member. Feasibility was determined by recruitment and ability to complete the study procedures in a pragmatic fashion that incorporated resident education. The primary outcome was accuracy of diagnoses, and secondary outcomes included number of tests and diagnoses generated, percent of “can't miss diagnoses mentioned,” speaking order and psychological reactions of group members.

**Results:**

Twenty-five (9 PGY-2, 8 PGY-3, and 8 PGY-4) of 29 eligible residents participated in the sessions. There was no significant difference in accuracy of diagnosis between the brain-writing and brainstorming cases (mean = 73% vs 72%, *p* = 0.51). Brainstorming cases listed significantly more tests (mean = 11.9 vs 9.6, *p* = 0.001) but not more diagnoses (mean = 9.60 vs 9.12, *p* = 0.07). Junior residents spoke later and verbalized significantly fewer diagnoses and tests than senior residents in both brainstorming and brain-writing groups. There was no statistically significant difference in psychological outcomes of junior and senior residents in each group.

**Discussion:**

It is feasible to examine the impact of a behavioral-based intervention among medical trainees in a small case-based learning environment. This study, limited by a small sample size, did not find that brain-writing improved decision quality.

## Introduction

Diagnostic errors contribute to an estimated 40,000 to 80,000 hospital deaths per year and within neurology, approximately 9% of ischemic strokes are misdiagnosed on presentation to the emergency department.^1-3^ Many of these errors are likely due to failures of thinking and decision making.^4-6^ There has been significant research regarding cognitive practices to improve decision making, but its application to medical education and practice has been limited.^7-10^

“Brain-writing” is a method described in the psychology literature, which entails members of a group writing down ideas individually to improve the idea-generation capability of the group.^11-14^ In contrast to brainstorming, where a group discusses ideas together, brain-writing is intended to address several common problems in group-based idea generation, particularly production blocking, evaluation apprehension, and free-riding. Production blocking refers to the diminished capability of an individual to generate ideas when their attention is devoted to listening to another person speak. Evaluation apprehension refers to the phenomenon by which individuals do not speak up out of fear of being negatively judged or disagreeing with a high-status member of the group. Free-riding refers to the process by which individuals abstain from participating, knowing that others will contribute, and feeling that their own contributions may not be important additions.^11-13,15-17^

Brain-writing was initially proposed by VanGundy in 1984 to improve the group-based development of new product ideas.^11^ Mullen et al.^17^ showed that individuals writing down ideas were more productive than group-based brainstorming. Furthermore, Paulus and Yang^16^ demonstrated the potential of group-based brain-writing to improve group-based decision making compared with separate individual idea generation.

Brain-writing has the potential to address these processes and improve the engagement of team members in clinical settings.^13^ This potential benefit of brain-writing may be conceptualized through the theoretical framework of Social Capital Theory, which has demonstrated the potential for characteristics associated with different levels of social capital, to impact educational experience and achievement.^18,19^ For example, senior residents, due to their seniority and knowledge-base, might participate more actively and have different educational experiences than junior residents.

Clinical decision making at Academic Medical Centers often takes place among a group involving attending physicians, fellows, and residents of different levels of training. Although the unique perspectives of these different individuals should theoretically help strengthen the diagnostic capability of the group, these perspectives may not be fully captured because of differences in power and status across group members that can magnify the aforementioned group decision-making problems.^20^ Brain-writing is one potential tool to capture the perspectives of all individuals in a clinical group-based setting regardless of power and status dynamics.

The objective of this study was to assess the feasibility of investigating whether brain-writing could improve group-based clinical decision making and the experience of the team among trainees in an Academic Medical Center.

## Methods

We conducted a repeated-measures feasibility study based on 6 clinical problem-solving sessions embedded within the neurology residency program at Columbia University Irving Medical Center. The sessions were held within a regularly occurring weekly morning block dedicated to resident education, involving all neurology residents participating in small groups over a rotating 6-week period. We decided on 6 sessions in a pragmatic fashion as to not interrupt future planned didactic sessions and to provide the opportunity for all neurology residents to be involved in the sessions. Given that the residency classes were relatively small, we did not collect demographic data such as age, sex, and race/ethnicity, as these data combined with PGY data could have posed a risk to the confidentiality of individual participants. The sessions were held over videoconference due to the COVID-19 pandemic.

In each session, groups of residents were presented with 6 separate clinical cases. The same 6 cases were presented to each group of residents. Residents were asked not to share the details of cases with residents scheduled for later sessions. The cases were constructed by the residency Program Director Dr. Michelle Bell and by Dr. Jeremy Ader, based on the New England Journal of Medicine (NEJM) Neurology case records. Cases published more than 3 years before the sessions were selected to decrease the likelihood that they would be familiar to residents participating in the intervention. The NEJM cases, as they exist, all outline a core differential diagnosis and a final diagnosis. Dr. Bell and Dr. Ader reviewed the cases and defined a set of “can't miss” diagnoses for each case. The cases and the corresponding “can't miss” diagnoses were discussed with 4 other core neurology teaching faculty. Expert consensus was established after the faculty did not have additional diagnoses to add to the “can't miss diagnoses” list. After proposal of the final list of “can't miss diagnoses,” there were no instances in which core faculty members felt that a proposed diagnosis should be removed.

Before the start of the sessions, residents were told by e-mail that participation in the exercise was part of a research study on a behavioral intervention designed to capture the diagnostic potential of all individuals in the group. They were told that videos of the sessions would be recorded, that at the end of the study they would receive a $50 gift card, and that participation was optional.

Each session was precepted by Dr. Bell. During each session, 3 cases were treated as brainstorming cases and 3 cases were treated as brain-writing cases. For brainstorming cases, the clinical vignette was read by Dr. Bell. Participants would then discuss the case and collectively develop a list of tests they would like to order as well as possible diagnoses and decide on a final diagnosis. For brain-writing cases, the clinical vignette was read by Dr. Bell. Before group discussion, participants were asked to individually write down a list of tests they would like to order and possible diagnoses through a Qualtrics survey with free-text question boxes. The group would then discuss the case and develop a list of tests they would like to order, possible diagnoses and a final diagnosis. Approximately 15 minutes were allotted per case, although there was no time-limit given to the brain-writing or brainstorming exercises, which all concluded when the residents felt that the group discussion was complete. For both the brainstorming and brain-writing groups, Dr. Bell recorded the list of possible diagnoses discussed as well as the final diagnosis.

Before the start of the sessions, cases were determined to be treated as brain-writing or brainstorming cases for different weeks such that each clinical case was treated as a brainstorming case for 3 sessions and a brain-writing case for 3 sessions. The weekly assignments were determined in such a way that there was a unique set of brainstorming and brain-writing cases for each session. Brainstorming cases were presented before brain-writing cases for half of the sessions, and on other weeks, brain-writing cases were presented first.

After the conclusion of each case, participants completed a set of survey questions. The authors designed the survey based on questions used in the psychology literature related to 4 elements of group dynamics— cohesion, psychological safety, process satisfaction, and confidence.^21-24^ For the brainstorming cases and brain-writing cases, participants were asked questions regarding how comfortable they were sharing their opinions, how much other group members appreciated what they had to say, how much friction there was in the group, their satisfaction with the group decision process, and their confidence with the group's diagnosis on 5-point Likert scales. Dr. Bell then revealed the accurate diagnosis and reviewed the cases and a set of teaching points with the residents. After the completion of the 6 cases, the residents participated in a lecture given by Dr. Adam Galinsky, Professor of Leadership and Ethics at the Columbia Business School, about decision-making science and its application to diagnostic reasoning.

After completing all 6 sessions, videos of the sessions were reviewed by Dr. Ader to determine the speaking order of the group according to level of training for each case. Dr. Ader also reviewed the videos to record the number of tests and diagnoses that were verbalized by each member of the group.

Based on existing literature of house staff diagnostic accuracy, we conducted a power calculation assuming 66% mean resident accuracy with a standard deviation of 10% and an anticipated absolute difference in mean accuracy of 6% between groups and an alpha of 0.05.^25^ We estimated we would need a sample size of 88 cases per group to detect a statistically significant difference between the groups. As previously discussed, for pragmatic purposes, we would only be able to conduct 18 brainwriting cases over a 6-week period.

The primary goal was to assess the feasibility of studying a behavioral intervention to improve group-based decision making, within a regularly occurring educational session in an academic neurology residency. Feasibility was determined by recruitment, ability to complete the study procedures and collect pertinent data in a practical manner, and ability to integrate educational components throughout the study.^26^

After assessment for feasibility, the primary outcome was the accuracy of the diagnosis, defined as whether the final diagnosis corresponded with the accurate diagnosis, compared between the brain-writing and brainstorming groups. Both accuracy and number of diagnoses generated reflect the potential of brain-writing to improve idea generation. However, given the greater relevance of accuracy of diagnosis to clinical practice, this was treated as the primary outcome. In addition to number of diagnoses, secondary outcomes included the differences between groups regarding the number of tests generated by the group, the percent of “can't miss” diagnoses mentioned, the speaking order and number of diagnoses and tests verbalized by members who differed in years of training, and the psychological reactions of group members.

For all analyses, linear mixed models were used to examine the relationship between variables. Linear mixed models control for the fact that residents discussed and responded to multiple cases during their session and that the intervention was randomized across cases. For group level comparisons (e.g., examining differences for responses generated at the group level), we included random effects at the group level and at the case level. For individual level comparisons (e.g., examining differences for responses generated at the individual level), we included random effects at the individual level and at the case level. If a model failed to converge or indicated singular fit, the random effect that explained the least variance was removed from the model to assuage the error.^27^ For specific between-group comparisons (e.g., PGY-2 residents compared to PGY-3 residents), we followed up these models with pairwise comparisons using Bonferroni correction. The *p* values are taken from these models, whereas the means reported are unadjusted means taken from the raw data. A *p* value cutoff of <0.05 was used to determine statistical significance.

### Standard Protocol Approvals, Registrations, and Patient Consents

All participants in the study provided electronic consent to participate. The study was approved by the Columbia University Irving Medical Center Institutional Review Board under Protocol AAAT9001. The study was funded by a $5,000 grant from the Virginia Apgar Academy of Medical Educators.

### Data Availability

Data not provided in the article because of space limitations may be shared (anonymized) at the request of any qualified investigator for purposes of replicating procedures and results.

## Results

### Idea Generation

Twenty-five (9 PGY-2, 8 PGY-3, and 8 PGY-4) of 29 eligible residents participated in the sessions (Table 1). Each of the 6 sessions involved a different group of 3–5 residents, with at least one representative from each postgraduate training year (PGY): PGY-2, PGY-3, and PGY-4.

**Table 1:** Postgraduate Year of Members in Each Group

	Group 1	Group 2	Group 3	Group 4	Group 5	Group 6
PGY-2 (n = 9)	1	1	3	2	1	1
PGY-3 (n = 8)	1	2	1	1	2	1
PGY-4 (n = 8)	2	1	1	1	2	1
Total (n = 25)	4	4	5	4	5	3

Abbreviation: PGY = postgraduate year

We found that it was practical to study a behavioral health intervention designed to improve diagnostic accuracy within a regularly occurring academic curriculum in a neurology residency program. We recruited 86 % of eligible neurology residents. We were able to complete exercises and run sessions without issue over videoconferencing software with integrated data collection through Qualtrics survey. In addition, we were able to conduct the study while integrating educational components throughout the exercise, in the form of reviews of key teaching points of the cases, and a lecture at the end of the exercise, led by AG about group-based decision making.

There was no significant difference in accuracy between brain-writing (mean = 73%, SD = 0.45) and brainstorming cases (mean = 72%, SD = 0.45, *p* = 0.51, Table 2). However, brainstorming cases listed significantly more tests (mean = 11.92, SD = 6.02) relative to the brain-writing cases (mean = 9.63, SD = 2.89, *p* = 0.001). There was no significant difference in the number of differential diagnoses listed by brainstorming cases (mean = 9.60, SD = 3.89) compared with brain-writing cases (mean = 9.12, SD = 2.79, *p* = 0.07).

**Table 2:** Idea Generation, Verbalization, and Speaking Order

	Brainstorming Mean (SD)	Brain-writing Mean (SD)	*p* Value
Accuracy	73% (45%)	72% (45%)	0.51
Percent of “can't miss” diagnoses	52% (21%)	56% (30%)	0.24
No. of diagnoses mentioned by group	9.60 (3.89)	9.12 (2.79)	0.07
No. of diagnoses verbalized by individuals	3.07 (3.09)	2.75 (2.52)	0.86
PGY-2	1.35 (1.23)	1.19 (1.59)	1.00
PGY-3	2.91 (2.39)	4.08 (2.48)	0.96
PGY-4	4.96 (3.93)	3.17 (2.53)	0.37
No. of tests mentioned by group	11.92 (6.02)	9.63 (2.89)	0.001
No. of tests verbalized by individuals	3.17 (3.71)	2.60 (2.38)	0.231
PGY-2	0.52 (0.79)	1.59 (1.95)	1.00
PGY-3	3.61 (3.45)	3.42 (2.76)	1.00
PGY-4	5.39 (4.15)	2.92 (2.06)	0.02
Order of speaking	2.58 (1.23)	2.64 (1.27)	0.84
PGY-2	3.04 (1.22)	3.26 (1.26)	1.00
PGY-3	2.43 (1.04)	2.21 (1.22)	1.00
PGY-4	2.26 (1.32)	2.38 (1.10)	1.00

Abbreviation: PGY = postgraduate year

There was no significant difference in the percentage of “can't miss” diagnoses listed by the brain-writing (mean = 56%, SD = 0.30) vs brainstorming groups (52%, SD = 0.21, *p* = 0.24). There was no significant interaction between whether brain-writing cases came before or after brainstorming cases.

During the brain-writing cases, there was no significant difference between the number of tests listed by PGY-2 residents (mean = 6.11, SD = 3.80) compared with PGY-3 residents (mean = 7.25, SD = 4.88, *p* = 1.00) or PGY-4 residents (mean = 6.88, SD = 4.57, *p* = 1.00). There was no significant difference in the number of diagnoses listed by PGY-2 residents (mean = 4.11, SD = 1.40) compared with PGY-3 residents (mean = 6.12, SD = 3.43, *p* = 0.11) or PGY-4 residents (mean = 5.08, SD = 2.30, *p* = 0.84). This analysis was not performed for the brainstorming group, in which residents did not individually list tests.

Cases discussed later in the sessions had significantly fewer diagnoses (*p* < 0.04) suggesting fatigue played a role.

### Speaking Order and Verbalization

Among both brainstorming and brain-writing cases, PGY-2 residents spoke later (mean = 3.16, SD = 1.23) than PGY-3 (mean = 2.32, SD = 1.12, *p* = 0.001) and PGY-4 (mean = 2.32, SD = 1.20, *p* = 0.001) residents in group discussions. This relationship between speaking order and years of experience did not significantly differ between the brain-writing and brainstorming cases (*p* = 0.64).

Among brainstorming and brain-writing cases, PGY-2 residents verbalized fewer tests (mean = 1.10, SD = 1.61) compared with PGY-3 (mean = 3.51, SD = 3.08, *p* = 0.012) and PGY-4 residents (mean = 4.13, SD = 3.46, *p* = 0.002). The relationship between verbalizing tests and years of experience was weaker in the brain-writing cases compared with the brainstorming cases such that in the brain-writing cases, the number of tests verbalized was the lowest for PGY-2 residents, then PGY-4 residents, and highest for PGY-3 residents, whereas in the brainstorming cases the number of tests verbalized followed a linear trend such that PGY-2 residents verbalized the least tests and then PGY-3 residents, followed by PGY-4 residents (*p* < 0.002).

Among brainstorming and brain-writing cases, PGY-2 residents verbalized fewer diagnoses (mean = 1.26, SD = 1.43) compared with PGY-3 (mean = 3.51, SD = 2.48, *p* = 0.013) and PGY-4 residents (mean = 4.04, SD = 3.37, *p* = 0.002). Similar to the case with verbalization of tests, the relationship between verbalization of diagnoses and years of experience differed across the brain-writing and brainstorming cases such that in the brain-writing cases, the number of diagnoses verbalized was the lowest for PGY-2 residents, then PGY-4 residents, and highest for PGY-3 residents, whereas in the brainstorming cases, the number of diagnoses verbalized followed a linear trend such that PGY-2 residents verbalized the least diagnoses and then PGY-3 residents, followed by PGY-4 residents (*p* < 0.009).

### Psychological Experience

There was no significant difference in confidence between residents in the brain-writing (mean = 4.15, SD = 4.15) vs brainstorming cases (mean = 3.84, SD = 0.84, *p* = 0.38, Table 3). There was no difference in confidence between PGY-2 and PGY-3 or PGY-4 residents, respectively, in the brain-writing (PGY-2 mean = 4.19, SD = 0.74; PGY-3 mean = 4.17, SD = 0.92, *p* = 1.00; PGY-4 mean = 4.08, SD = 0.65, *p* = 1.00) or brainstorming cases (PGY-2 mean = 3.67, SD = 0.88; PGY-3 mean 3.79, SD = 0.88, *p* = 1.00; PGY-4 mean = 4.08, SD = 0.58, *p* = 1.00).

**Table 3:** Psychological Outcomes

	Mean of 5-point Likert scale (SD)	Mean of 5-point Likert scale (SD)	*p* Value
How comfortable did you feel sharing your opinion?	3.44 (1.5)	3.64 (1.33)	0.28
PGY-2	3.26 (1.40)	3.11 (1.31)	1.00
PGY-3	3.38 (1.61)	3.79 (1.32)	1.00
PGY-4	3.71 (1.52)	4.08 (1.21)	1.00
How much did the other group members appreciate what you had to say?	3.11 (0.65)	3.09 (0.68)	0.85
PGY-2	2.96 (0.59)	2.93 (0.62)	1.00
PGY-3	3.08 (0.65)	3.17 (0.70)	1.00
PGY-4	3.29 (0.69)	3.21 (0.72)	1.00
How much friction was there in your group?	1.09 (0.29)	1.04 (0.20)	0.06
PGY-2	1.04 (0.19)	1.04 (0.19)	1.00
PGY-3	1.21 (0.41)	1.08 (0.28)	0.39
PGY-4	1.04 (0.20)	1.00 (0.00)	1.00
How satisfied are you with how this decision was made?	4.21 (0.68)	4.23 (0.63)	0.77
PGY-2	4.22 (0.64)	4.44 (0.64)	1.00
PGY-3	4.21 (0.83)	4.08 (0.65)	1.00
PGY-4	4.21 (0.59)	4.12 (0.54)	1.00
How confident are you with the group's diagnosis?	3.84 (0.81)	4.15 (0.77)	0.39
PGY-2	3.67 (0.88)	4.19 (0.74)	0.96
PGY-3	3.79 (0.88)	4.17 (0.92)	1.00
PGY-4	4.08 (0.58)	4.08 (0.65)	1.00

Abbreviation: PGY = postgraduate year.

There was no significant difference in satisfaction between residents in the brain-writing (mean = 4.23, SD = 0.63) vs brainstorming cases (mean = 4.21, SD = 0.68, *p* = 0.77). There was no difference in satisfaction between PGY-2 and PGY-3 or PGY-4 residents, respectively, in the brain-writing (PGY-2 mean = 4.44, SD = 0.64; PGY-3 mean = 4.08, SD = 0.65, *p* = 1.00; PGY-4 mean = 4.12, SD = 0.54, *p* = 1.00) or brainstorming groups (PGY-2 mean = 4.22, SD = 0.64; PGY-3 mean = 4.21, SD 0.83, *p* = 1.00; PGY-4 mean = 4.21, SD = 0.59, *p* = 1.00).

There was no difference in resident level of comfort in brain-writing (mean = 3.64, SD = 1.33) compared with brainstorming cases (mean = 3.44, SD = 1.50, *p* = 0.28). There was no difference in comfort between PGY-2 residents (mean = 3.19, SD = 1.35) compared with PGY-3 (mean = 3.58, SD = 1.47, *p* = 1.00) or PGY-4 residents (mean = 3.90, SD = 1.37, *p* = 0.46).

There was no difference regarding the amount of friction that residents perceived during the brain-writing (mean = 1.04, SD = 0.20) compared with the brainstorming cases (mean = 1.09, SD = 0.29, *p* = 0.06). There was no difference in the level of friction perceived by PGY-2 (mean = 1.04, SD = 0.19), compared with PGY-3 residents (mean = 1.15, SD = 0.36, *p* = 0.22), or PGY-4 residents (mean = 1.02, SD = 0.14, *p* = 1.00).

There was no difference in the degree to which residents felt appreciated in the brain-writing (mean = 3.09, SD = 0.68) compared with the brainstorming cases (mean = 3.11, SD = 0.65, *p* = 0.85). There was no difference in perceived appreciation between PGY-2 residents (mean = 2.94, SD = 0.60) compared with PGY-3 residents (mean = 3.12, SD = 0.67, *p* = 1.00) and PGY-4 residents (mean = 3.25, SD = 0.70, *p* = 0.80).

## Discussion

This study suggests that it is feasible to examine a behavioral health intervention in a case-based educational setting within a neurology residency. Our study had strong recruitment, was conducted seamlessly within a regularly occurring educational session without issue, and integrated education throughout the exercise.

The study was underpowered to detect significant differences between the brain-writing and brainstorming groups. The completion of this study required use of time and resources that were dedicated to resident education. Given that study feasibility and ability to incorporate resident education were unproven at the start of this study and the neurology residency had existing educational plans, we could not devote more than 6 resident education sessions to this study. This allowed us to complete 18 cases per group. We estimated that 88 cases per group would be required to detect a statistically significant difference in diagnostic accuracy between groups. Given the feasibility of this study, future studies may consider devoting additional time and resources to increase the number of cases and achieve power to detect differences between groups.

We did not find evidence that brain-writing improves the quality of decisions in a clinical group. However, our study suggests that the practice may influence the generation of ideas and provides nonstatistically significant directional evidence that the practice may influence trainees' psychological experience.

More diagnoses were discussed in brainstorming cases than in brain-writing cases, although this was not statistically significant. This ran counter to our expectation that the brain-writing groups, by first independently generating lists of tests and diagnoses, would discuss a larger number of ideas as a group. Our finding might be explained by brainstorming case participants generating ideas “out loud,” whereas the brain-writing groups generated ideas through “brain-writing” and only chose to discuss the ideas they felt merited group attention. The degree of accuracy between the 2 groups was similar, and the brain-writing groups, despite discussing fewer tests and diagnoses, mentioned a slightly higher percentage of “can't miss” diagnoses, although this difference was not statistically significant. This raises the possibility of more focused discussions in the brain-writing group, highlighting a possible mechanism through which brain-writing might improve the efficiency of group decision making. Such a mechanism might assuage potential concerns about brain-writing adding time to the decision-making process in the clinical setting. We did not measure the length of time of the discussions, although future studies could consider collecting this data, given the potential impact of behavioral interventions to improve the efficiency of group-based discussions.

Brain-writing did not seem to affect the participation of junior residents. We found that junior residents spoke later in the discussion than senior residents, and this finding did not differ between brain-writing and brainstorming cases. Junior residents also verbalized fewer tests and diagnoses than senior residents. This relationship between resident experience and degree of verbalization was weaker among the brain-writing group regarding verbalization of tests but stronger regarding verbalization of diagnoses.

We found nonstatistically significant directional evidence that residents were more confident, comfortable, and satisfied in the brain-writing cases compared with the brainstorming cases. There was also nonstatistically significant directional evidence that junior residents were more confident and satisfied than their senior residents during brain-writing cases compared with the brainstorming groups. One potential explanation for these reactions is that residents in the brain-writing group, and junior residents, in particular, may have felt more engaged in the decision-making process. This speaks to the potential for brain-writing to invite all trainees to intellectually engage in a discussion and overcome the common group decision-making problems discussed above.^11-13,15-17^ Through this process, brain-writing could potentially improve the educational engagement and experience of participants in a case-based problem-solving group.

Cases discussed later in sessions had fewer diagnoses, which likely speaks to participant fatigue. Although this is not an unsurprising finding, it does support the common clinical practices of discussing new patients early in the course of clinical rounds—as the review of new patients' history, workup, and diagnoses often require a particularly high cognitive load.

Medical trainees are taught to give standardized patient presentations which consist of a single person presenting a series of data, starting with the history of present illness, and culminating in the assessment and plan. This format has been the presumed ideal organizational framework for consolidating clinical data and developing a differential and ultimately a most-likely diagnosis.^28^ In Academic Medical Centers, this presentation is often delivered to clinicians of different backgrounds and levels of training. As the group receives information, it must also process the information, and at some point, during or after the presentation, generate ideas and participate in critical discussion to improve the differential diagnosis and plan. However, the way group members generate and discuss ideas may influence the quality of the differential diagnosis.

For example, having the presenter suggest a diagnosis may introduce biases that focus the conversation and attention around a particular idea, at the expense of other ideas. In addition the biases discussed above,^11-13,15-17^ other biases include premature closure, order effects, diagnostic momentum, framing effects, and confirmation bias.^29^

Our study demonstrated the feasibility of evaluating the impact of brain-writing in a clinical and educational setting within an Academic Medical Center and suggests the potential for brain-writing to impact idea generation and participant experience. Although additional higher-powered studies are needed, it is possible that brain-writing, while potentially of benefit in creative processes such as product development, might not have the same benefit in group clinical diagnosis. Our study, while specifically demonstrating the feasibility of examining brain-writing in a clinical educational setting, also suggests the potential to similarly study other behavioral science interventions from the psychology literature that might improve group diagnostic capability, engagement of junior members, and educational experience.

The study had several limitations. First, as previously mentioned, given the limited size of this study, it did not have enough statistical power to detect the effect of brain-writing on the decision-making process and group experience. Future studies based on the design of this study need a greater number of residents and/or more clinical problem-solving sessions to detect these effects. Second, it is possible that some residents were familiar with the cases before their sessions. We attempted to minimize this possibility by choosing NEJM cases that were older than 3 years and by asking residents not to discuss the cases with residents participating in later sessions. Third, the group make-up differed from session-to session regarding the number of residents and the distribution of residents among years of training. However, we ensured that every session involved at least one representative from each year of neurology training. Fourth, given the circumstances of the COVID-19 pandemic, this feasibility study was conducted over videoconference and therefore may not reflect the feasibility of testing this intervention in-person or the impact that it may have had in-person. However, given the growing presence of videoconferencing in medical education and clinical care, this is an important setting for evaluating behavioral science interventions.

This study demonstrated the feasibility of examining the impact of a behavioral science intervention among medical trainees in a small case-based learning environment. The intervention, based on the technique of brain-writing, consisted of individuals in the group writing down their ideas prior to a group discussion. The potential benefit of brain-writing is to better engage all members of a group in decision making to improve decision quality and group experience. The study was limited by a small sample size and did not suggest any impact of brain-writing on decision quality, although it may have affected idea generation. In addition to demonstrating the feasibility of studying brain-writing, our study also suggests the potential to similarly study other behavioral science interventions that may affect clinical decision making and educational experience.

## Glossary

PGY: postgraduate year
